# The Change of Expression Configuration Affects Identity-Dependent Expression Aftereffect but Not Identity-Independent Expression Aftereffect

**DOI:** 10.3389/fpsyg.2015.01937

**Published:** 2015-12-22

**Authors:** Miao Song, Keizo Shinomori, Qian Qian, Jun Yin, Weiming Zeng

**Affiliations:** ^1^College of Information Engineering, Shanghai Maritime UniversityShanghai, China; ^2^School of Information, Kochi University of TechnologyKochi, Japan; ^3^Yunnan Key Laboratory of Computer Technology Applications, Kunming University of Science and TechnologyKunming, China

**Keywords:** facial expression, adaptation, aftereffect, visual representation, vision

## Abstract

The present study examined the influence of expression configuration on cross-identity expression aftereffect. The expression configuration refers to the spatial arrangement of facial features in a face for conveying an emotion, e.g., an open-mouth smile vs. a closed-mouth smile. In the first of two experiments, the expression aftereffect is measured using a cross-identity/cross-expression configuration factorial design. The facial identities of test faces were the same or different from the adaptor, while orthogonally, the expression configurations of those facial identities were also the same or different. The results show that the change of expression configuration impaired the expression aftereffect when the facial identities of adaptor and tests were the same; however, the impairment effect disappears when facial identities were different, indicating the identity-independent expression representation is more robust to the change of the expression configuration in comparison with the identity-dependent expression representation. In the second experiment, we used schematic line faces as adaptors and real faces as tests to minimize the similarity between the adaptor and tests, which is expected to exclude the contribution from the identity-dependent expression representation to expression aftereffect. The second experiment yields a similar result as the identity-independent expression aftereffect observed in Experiment 1. The findings indicate the different neural sensitivities to expression configuration for identity-dependent and identity-independent expression systems.

## Introduction

One key issue in face study is to understand how emotional expression is represented in the human visual system. According to the classical cognitive model (Bruce and Young, 1986) and neural model (Haxby et al., 2000), emotional expression is consider to be represented and processed independent of facial identity. This view is supported by several lines of evidence. First, neuropathological studies have reported that some brain injury patients exhibit selective impairments of ability to recognize expression but not face, or vice versa (e.g., Bruyer et al., 1983; Young et al., 1993; Palermo et al., 2011), indicating a double dissociation between the representations of identity and expression. Second, early behavior studies found no difference in reaction times when making expression matching decisions to familiar and unfamiliar faces (e.g., Bruce and Young, 1986; Ellis et al., 1990), suggesting that expression processing does not depend on the familiarity of facial identity. Finally, single-unit recording and fMRI studies have found that expression may be processed in the superior temporal sulcus, located in lateral occipitotemporal cortex, whereas the process of facial identity preferentially involves the face fusiform area (FFA), located in inferior occipitotemporal cortex (e.g., Hasselmo et al., 1989; Haxby et al., 2000; Winston et al., 2004).

Despite substantial evidence supporting independent representation of expression, there are a growing number of studies suggesting that expression representation is related to facial identity. Using a Ganer's speeded-classification task, variation in identity was found to interfere with the performance of expression classification (Schweinberger and Soukup, 1998; Schweinberger et al., 1999) or vice versa (Ganel and Goshen-Gottstein, 2004, for a recent further discussion, see Wang et al., 2013). On the other hand, happy expression was reported to improve the familiarity ratings of face (Baudouin et al., 2000) and the identity recognition speed (e.g., Kaufmann and Schweinber, 2004). Furthermore, Chen et al. (2011) compared effects of all basic facial expressions on identity recognition, and observed the different reaction times when faces are matched in different expression conditions, indicating the modulation of expression on the processing of face identity. In fact, besides emotional expression in face, body expressions and emotional cues in the background scene could also affect the processing of facial identity (Van den Stock and de Gelder, 2012, 2014), suggesting an general interaction mechanism underlying the processing of emotion and facial identity. In support of these behavior studies, single-cell studies have identified certain cells in monkey STS area (e.g., Sugase et al., 1999) and amygdale area (e.g., Gohard et al., 2007) which are responsive to both identity and expression stimuli, indicating that representations of emotional expression and facial identity may be overlapped.

In fact, concerning the relationship between expression and identity representations, besides the radical view of complete independence or the opposing view of highly dependence, there is also a third view to describe representation of expression, i.e., there are two types of expression representations, one depends on facial identity while the other is independent of facial identity. In a pioneer study, Fox and Barton (2007) have provided the evidence for this view of partial dependence, using the cross-identity expression adaptation. Adaptation refers to the phenomenon that prolonged exposure to a given stimulus would result in a subsequent perceptual bias (i.e., aftereffect). For instance, after viewing a unidirectional moving stimulus, an observer will perceive a subsequent stationary stimulus as moving in the opposite direction. As visual adaptation can reflect short-term responsiveness changes in the human neural system, it has been well developed to study the processes of low-level visual properties (for review see Durgin and Proffitt, 1996; Anstis et al., 1998; Clifford, 2002), and complex visual stimuli, such as various facial dimensions (e.g., Leopold et al., 2001; Zhao and Chubb, 2001; Rhodes et al., 2003; Hsu and Young, 2004; Yamashita et al., 2005, for review see Webster et al., 2004; Webster and MacLeod, 2011). As far as expression adaptation are concerned, within a series of ambiguous expression images morphing between two expressions (e.g., happy and angry faces), adaptation to one expression (e.g., happy face) will distort the perception of observers to facilitate perceiving the ambiguous images as the other expression (i.e., angry expression). The magnitude of expression aftereffect can be indexed by the differences in response proportions of subjects before and after adaptation.

In Fox and Barton's adaptation experiments, expression aftereffect is measured using adaptors and tests that were both the same and different identities. The expression aftereffect is strongest when the identities of the adaptor and test faces were the same, however, it is still present, but much reduced when the adaptor and test faces differ in facial identity. These results were interpreted as evidence that there are two different neural representations in expression system, i.e., identity-dependent and identity-independent expression representations. If the adaptor and test faces are congruent, both identity-dependent and identity-independent expression representations are adapted and contribute to the expression aftereffect; if the adaptor and test faces are incongruent, only identity-independent expression representation contributes to the expression aftereffect. This explains why the expression aftereffect in incongruent condition is much weaker than that in congruent condition.

In support of Fox and Barton, several subsequent works replicated this cross-identity expression adaptation with a variety of experimental paradigms and stimuli (Ellamil et al., 2008; Campbell and Burke, 2009; Vida and Mondloch, 2009; Pell and Richards, 2013). The cross-identity expression aftereffect was found to occur generally in five basic expressions with approximately the same extent of transfer, suggesting different expression representations depend on identity in a similar way (Campbell and Burke, 2009). A recent study further reported that the overlapping expression representations (e.g., the representation for similar emotional features of disgusted and angry faces) are also identity-dependent, it was found that the adaptation to a disgusted face would bias perception away from angry face and this cross-emotion expression adaptation generalizes across identity (i.e., Pell and Richards, 2013). Additionally, Vida and Mondloch (2009) shows that children (5–9-year-olds) display an adult-like transfer of expression aftereffect across identities.

The above studies provided strong evidence for the existence of identity-dependent and identity-independent expression representations, little is, however, known about the nature of these two different types of expression representations, especially about their sensitivity to expression configuration. Expression can be viewed as stereotyped geometrical variations in facial configurations that correspond to well-defined action patterns (Webster and MacLeod, 2011). Although there is only a small set of six basic expressions, one expression can be conveyed with different varieties of expression configurations within an emotion category to present subtle emotional information (Rozin et al., 1994). For instance, humans can open their lips and show their teeth to express a high-intensity smile, or they can simply crinkle the corner of their eye to express a weak-intensity smile. So far, there is relatively little knowledge regarding the sensitivity to expression configuration of the identity-dependent and identity-independent expression representations, this issue is, however, helps to obtain a better understanding for the processing mechanism of the human expression system, as well as for an understanding for the functional difference between the identity-dependent and identity-independent expression systems.

The expression aftereffect is an effective measure to assess the sensitivity of expression system to expression configuration. Fox and Barton (2007) have showed that the magnitude of expression aftereffect was almost not affected when the adaptor was changed to a picture of the same expression in the same individual but that differs from the image used to create the morphed test stimuli, indicating that the expression aftereffect is not sensitive to the subtle change in the expression configuration to some extent. However, the different expression images of the same expression by the same person are still highly similar; this finding cannot well exclude the influence of expression configuration on expression aftereffects. In contrast, several other studies suggested that the expression aftereffect depends on the expression configuration. Butler et al. (2008) showed that the expression aftereffect can be produced by emotional facial features, but not by the same emotional features in a hybrid face with inconsistent expression configurations. On the other hand, Skinner and Benton (2010) reported that adaptation to the anti-expression, which is defined by the physically complementary expression configuration, would induce a significant expression aftereffect on the original emotional expression matching to the anti-expression (see also Cook et al., 2011; Juricevic and Webster, 2012). As the anti-expression does not convey the emotional information, this observation also reflects that the expression aftereffect is closely related to the spatial configuration of expression.

Although the previous studies have provided the cue about how expression configuration influences expression aftereffect, there is no studies directly examining this issue in a framework comprising identity-dependent and identity-independent elements. This is important because the identity-dependent and identity-independent expression representations may reside in different brain areas (Fox and Barton, 2007), and have different sensitivities to expression configuration (Harris et al., 2012, 2014). Morris et al. (1996) and Thielscher and Pessoa (2007) have previously reported that responses in the amygdala can be modulated by changes in the emotion's intensity of expression images, suggesting that the amygdala contain neuron population that tuned to the expression configuration. In contrast, in the recent studies, Harris found that amygdala area is relatively robust to expression change within the same emotion category in comparison to posterior superior temporal sulcus (pSTS), using static expression images (Harris et al., 2012) or dynamic movie as stimuli (Harris et al., 2014). Although the results from above studies are not completely consistent, the current evidence indicates that the brain areas may have the different neural sensitivities to the expression configuration.

In current study, we used the cross-identity expression adaptation (Fox and Barton, 2007) to investigate the sensitivities of identity-dependent and identity-independent expression representations, respectively. The experimental logic for adaptation is as follows: Given that the adaptation with the repedition of the completely same stimulus would reduce the neural response and induce the aftereffect to the maximum extent, the reduction of aftereffect should be observed when a particular dimension of stimulus is changed, provided that the underlying neural system codes that dimension. The aftereffect would become weak because the altered stimulus activates a new, non-adapted neural representation. In contrast, the aftereffect would remain the same if the underlying neural system is insensitive to differences along the altered stimulus dimension (Grill-Spector and Malach, 2001). As far as our research question is concerned, if the identity-dependent neural representation is sensitive to the expression configuration, the identity-dependent expression aftereffect should be reduced when the adaptor is changed to a expression configuration different from the tests; if identity-dependent neural representation is regardless of the processing of expression configuration, the expression aftereffect should be robust to the change in the expression configuration. The logic for identity-independent expression representation is similar.

Two experiments were performed in this study. In Experiment 1, we measured the expression aftereffect in an orthogonal 2 × 2 design, manipulating whether the adaptor and test faces exhibited the same or different facial identities, and independently, whether the expression configurations on the identities were the same or different. Experiment 1 thus includes the following 2 × 2 combinations (same or different: facial identity and expression configuration between adaptor and test faces): same identity/same configuration, same identity/different configuration, different identity/same configuration, different identity/different configuration. Given this factorial design, if identity-dependent and identity-independent expression representations have similar neural sensitivities to expression configuration, it is expected to observe that expression configuration change has similar effect on expression aftereffect regardless of the identities of adaptors and tests. In other word, there should be no the significant interaction between facial identity and expression configuration. On the contrary, if the sensitivities of these two expression representations are different, the interaction is expected to be observed. More specifically, if identity-dependent expression representation is sensitive to expression configuration, the expression aftereffect in same identity/different configuration condition should be weaker than that in the same identity/same configuration condition, otherwise, the aftereffect size in the two conditions should be approximately the same. Similarly, if identity-independent expression aftereffect is sensitive to expression configuration, the expression aftereffects in different identity/different configuration is expected to be weaker than that in different identity/same configuration condition, otherwise, we expect to observe the approximately same aftereffect in these two conditions.

In Experiment 2, we repeated the different identity/different configuration experimental condition using schematic line face as the adaptor and real faces as tests, which minimized the similarity in face features between adaptor and tests. Using these dissimilar stimuli, interference is expected to be excluded from the identity-dependent expression representation, and the effect of expression configuration on identity-independent expression representation to the expression aftereffect can be accurately evaluated.

## Experiment 1

The expression aftereffect is measured in the following way. We created morph series using two expression images of the same person. The images in the middle range of the morph series thus showed a recognizable identity but an ambiguous expression cue. After adapted to one expression, the subject was instructed to judge which expression the ambiguous morphing expression resembled. Generally, adaptation to one expression increased the possibility the ambiguous expression was judged as the other expression.

Experiment 1 included 4 adapting conditions as described in the introduction part, in which test faces were always the same among conditions, but the identity and/or the expression configuration of the adaptor were manipulated to create the different adapting conditions. The four conditions are respectively termed as SI/SC, SI/DC, DI/SC, and DI/DC, where the first two letters indicates whether adaptor and test face are of the same identity (SI: Same Identity) or not (DI: Different Identity), and last two letters indicates whether the expression configuration of adaptor are the same with that of test face (SC: Same Configuration) or not (DC: Different Configuration). For instance, SI/SC refers to the condition in which both the identity and the expression configuration of adaptor are identical to those of test face, whereas DI/DC means that adaptor differentiates with the test face in both identity and expression configuration.

### Subject

The subjects were sixteen paid students (five from Kochi University of Technology and eleven from Shanghai Maritime University, mean age: 19.6, *SD* = 2.8), and each subject completed eight 40-min sessions. All subjects have normal or corrected-to-normal vision, and were naïve to the purpose of the experiment. Naivety was confirmed during the debriefing that took place once they had finished. The protocol of two experiments was approved by the review boards of the Chinese Ethics Committee of Registering Clinical Trials, and informed consent was obtained in accordance with the principles in the Declaration of Helsinki.

### Stimuli and apparatus

The face stimuli used in Experiment 1 were selected from the affiliated image set of the Facial Action Coding System (Ekman et al., 2002) and the Cohn-Kanade AU-Coded Facial Expression Database (Kanade et al., 2000). The expression images in the two databases are coded by Face Action Coding System (Ekman and Oster, 1979; Ekman et al., 2002) and given an emotion label, which enables us to select same expression configuration for different photographic subjects in terms of their emotion labels.

**Figure 1:**
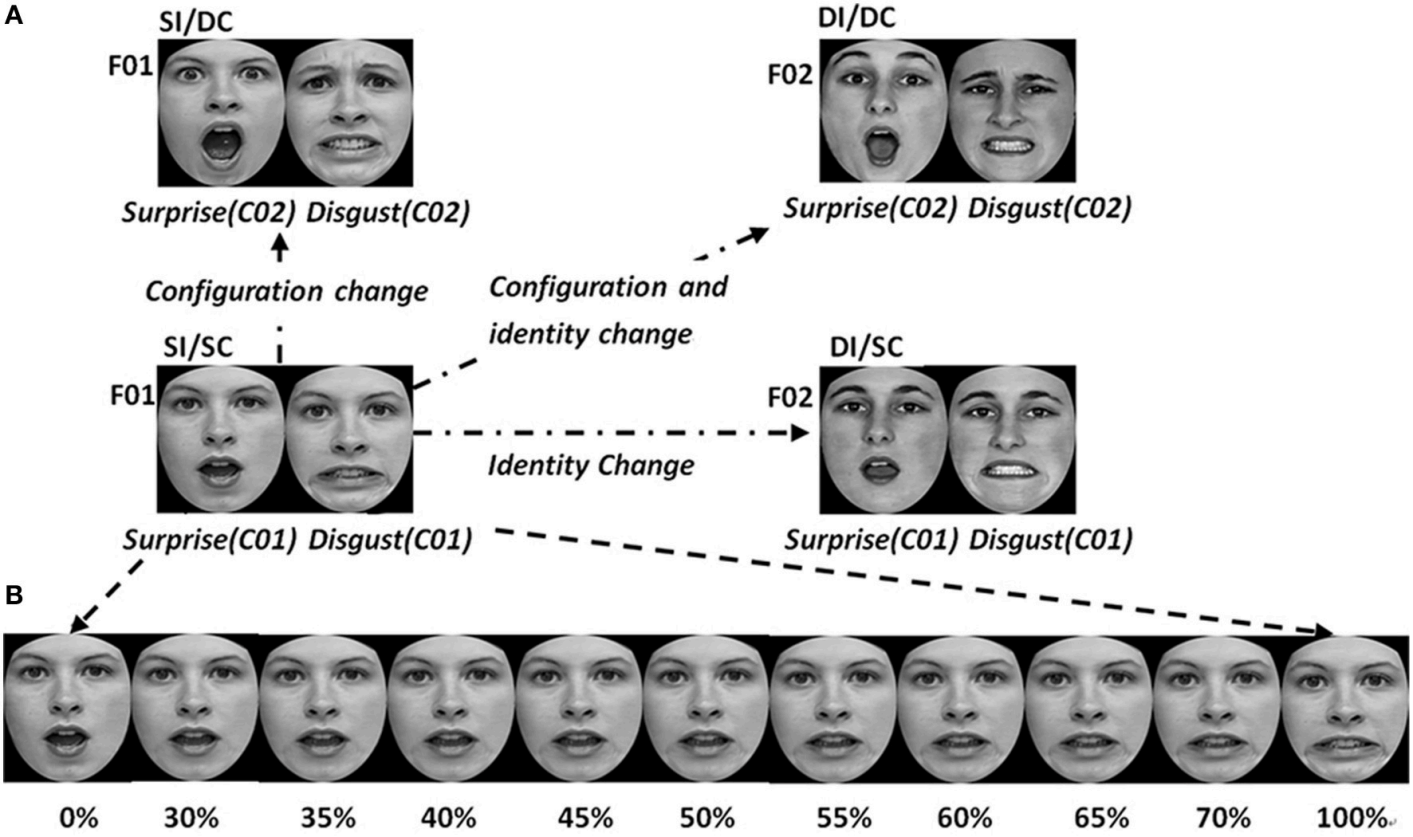
**Examples of the face stimuli used in experiment 1. (A)** The adaptors in four adapting conditions. **(B)** The surprised-disgusted expression pair as tests, which were created by morphing between two adapting faces used in the SI/SC condition. These test stimuli were always the same for four adapting conditions.

Happy, angry, surprised, and disgusted expressions were used in Experiment 1, which constituted two expression pairs, i.e., happy-angry and surprised-disgusted expression pairs. Illustrated by the case of surprised-disgusted expression pair, we selected two female photographic subjects (F01 and F02) depicting two expression configurations (C01 and C02) of surprise and disgusted expressions, resulting in four combinations of identity and expression configuration (F01 with C01, F01 with C02, F02 with C01, and F02 and C02) (see Figure 1A). The argument to select these photographic subjects is that they posed the most easily recognizable and high intensity expressions in the databases. The same method was used to select the adaptors of happy-angry expression pair, except that the two photographic subjects showing happy and angry expressions were male.

Using two expression images of the same photographic subject, we morphed a series of test faces using Abrosoft FantaMorph 5.0 for surprised-disgusted and happy-angry expression pairs, respectively, with the expression strength from 0 to 100% in steps of 5% in terms of the scale of the morphing software. The nine middle ambiguous images, which varied from 30 to 70%, served as test stimuli (see Figure 1B).

We used the same test faces but different adaptors in the four experimental conditions. As shown in Figure 1, the test faces were always the ambiguous expression images morphed between F01 with surprised expression (C01) and F01 with the disgusted expression (C01). In the same identity/same configuration condition, the same images used to construct the test faces were used as the adaptors (i.e., F01 with surprised expression C01, and F01 with disgusted expression C01). In the same identity/different configuration condition, the adaptors were the face images that had the same identity but expression configuration that differed from the test faces (i.e., F01 with the surprised expression C02, and F01 with disgusted expression C02). In different identity/same configuration condition, we used the face images that had different identities but the same expression configuration with test faces as the adaptors (i.e., F02 with surprise expression C01, and F02 with disgusted expression C01). Finally, in different identity/different configuration condition, the adaptors were the face images with different identities and expression configuration that differed from test faces (i.e., F02 with surprise expression C02, and F02 with disgusted expression C02).

All face stimuli used in Experiment 1 were cropped with an oval frame (leaving only internal features and external jaw), resized to 400^*^550 pixels, and set on a black background. Distinguishing features, such as moles or scars, were removed with the Spot Healing Brush tool. Luminance and contrast were manually adjusted to be comparable across all face images. The experiments were run with Cogent Psychophysics Toolbox extensions, and the visual stimuli were presented on a 19-inchLCD controlled by a DELL computer, with a vertical refresh rate adjusted to 85 Hz and a spatial resolution set to1024^*^768 pixels. Subjects were seated to view the monitor from the distance of 50 cm, and the visual stimuli subtended a visual angle of approximately 12° (horizontally) by 15° (vertically) in this distance.

To better control the expression configuration, we chose Caucasian faces from two FACS coded facial expression databases. The subjects were, however, Asian persons. One might doubt whether the other race effect could influence the recognition performance of subjects or pollute the data. We argue the other race effect would not be a main confounding factor for the following reasons. First, as the experimental task is to recognize facial expression rather than facial identity. It is well established that six basic expressions are generally regarded as universal cross different races (Ekman, 1993), and observers can reliably discriminate among the six basic facial expressions on other race faces (Ekman, 1993; Biehl et al., 1997; Lee et al., 2011). Although other studies also show that participants could recognize facial expression of a person of their own race better than of a person of another race (Izard, 1971; Ducci et al., 1982), this race effect generally occur when a large number of subtle expressions or micro-expressions are used as tests. In current experiment, only two expression images were used in each condition, and we carefully selected the easily recognizable and high intensity expressions images. The recognition test showed that the subjects can easily recognize the expression with 100% correct rate in these selected expression images. Finally, to confirm this further, we ran a pilot experiment by six subjects to compare the just noticeable differences (JNDs) of happy-angry expression pair of two Caucasian faces used in our experiment, with those of two Asian faces^1^. The results did not show a significant difference between these two conditions, indicating an approximately identical sensitivity of subjects to expression perception in Asian and Caucasian faces.

### Procedure

Each subject was tested individually. To familiarize them with the expression images used in the experiment and the experimental procedure, subjects were given oral instruction and short training blocks. Training stimuli included the unaltered version (0, 100%, also used as adaptor in the main experiment) plus two morphed versions (30, 70%, also used as tests in the main experiment) for happy-angry and disgusted-surprised expression pairs. A training block of eight trials was performed to help the subjects make the correct association between the expression and the corresponding button for response, and this was repeated until subjects succeeded to reach the 100% correct ratio.

Each trial consisted of an adaptor image presented for 4 s, followed by the test face for 200 ms (after a 100 ms noise mask). Time parameters were selected to obtain strong aftereffects based on the study of the dynamics of facial adaptation (Leopold et al., 2005). After the presentation of the test image, subjects performed a two-alternative forced choice (2-AFC) task to classify the presented image into one of two categories (i.e., two expression images used to create the morphed series). Feedback for pressing each button was given after each trial to confirm subjects' button responses. The subjects were instructed to attend to the face stimuli, but no fixation point was given. This is to prevent subjects from overly attending to local facial features near the fixation point.

**Figure 2:**
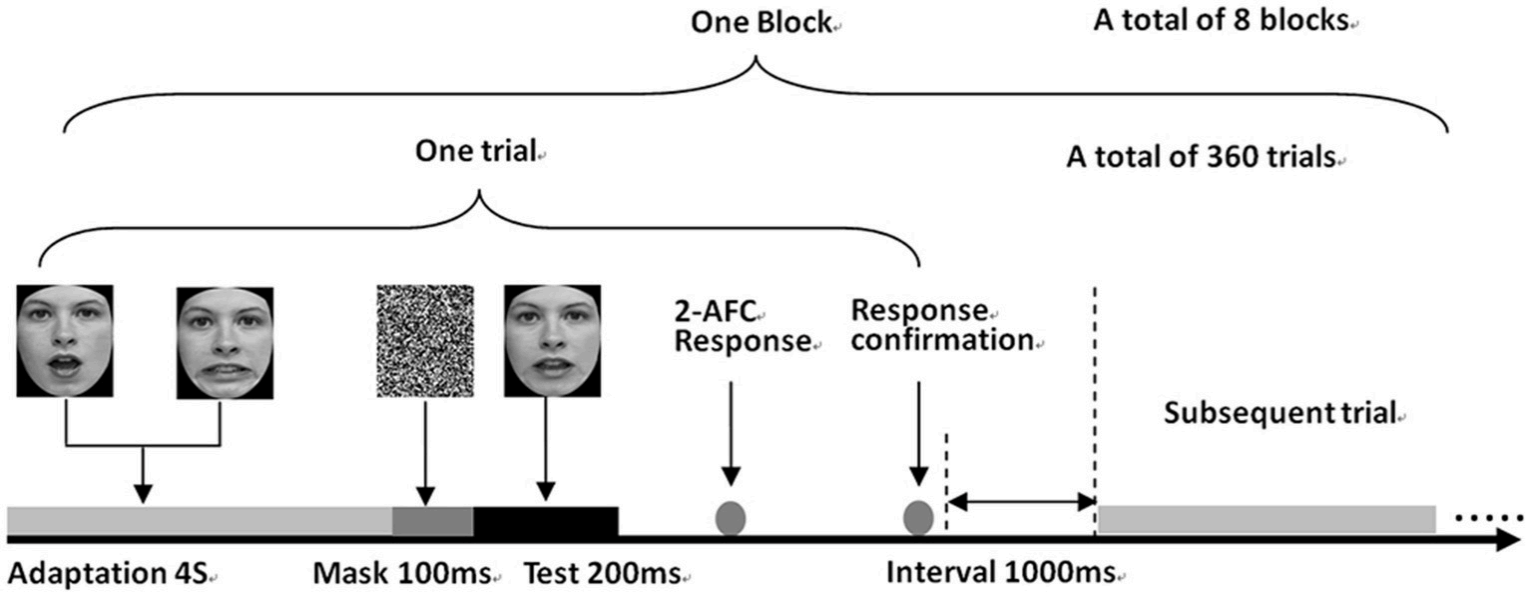
**Illustration of the visual adaptation and experimental procedure in Experiment 1**.

There were 20 trials for each test face, resulting in 360 (9 test face ^*^ 2 adaptors ^*^ 20 trials) trials per block. The experiment consisted of eight blocks, each for one of eight experimental conditions (2 expression pairs × 4 experimental conditions), performed in an order that was randomized across subjects. The trials for different tests in a block were also randomized. The duration of one block is approximately 40 min. In order to reduce fatigue, the subjects were allowed take a 5 min break after every 15-min experiment, they were also encouraged to take a break whenever they feel tired. The subjects participated in one block every other day and finished all experiments within 16 days. Figure 2 shows the procedure of Experiment 1.

### Data analysis

For each condition, we first determined the response proportion given for one of two choices (e.g., how many times does the subject respond “surprised”) at each test level for each subject. Then, we fit the data of the response proportion based on the maximum likelihood fitting procedure (Meeker and Escobar, 1995) using a logistic function formula as follows:

$$F(x=\alpha ;\alpha ,\beta )=\frac{1}{1+\text{exp}(-\beta (x-\alpha ))}$$

Where *x* is the morphing strength, *F*(*x*) is the probability of response, parameter α corresponds to the point of subjective point [PSE, *F*(*x* = α; α, β) = 0.5], and parameter β determines the slope of psychometric function. From these fits, the aftereffect magnitude was quantified as the difference (in morphing strength) between each subject's PSE adapted after one expression in a pair (e.g., happy expression) and that after the other expression (i.e., angry expression).

All statistical analyses were run on SPSS 19.0 software, and significance levels for all tests were set at *p* < 0.05. As the Kolmogorov-Smirnov normality test shows that there were no data violating the assumption of normality, we performed a three-way repeated measures ANOVA with the PSE difference of each subject as the dependent variable, facial identity (2 levels, same or different between adaptor and tests), expression configuration (2 levels, same or different between adaptor and tests), and expression type (2 levels, happy-angry expression pair or disgusted-surprise expression pair) as within-subject factors. Significant main effects and interactions were followed up with simple effect analyses, respectively. Paired samples t-tests were run on each condition to determine whether that condition generated a significant aftereffect, with the PSE adapted after one expression in a pair (e.g., happy expression) and that after the other expression (i.e., angry expression) for the same subject as paired variables. Finally, the *post-hoc* power analyses was performed for Three-way repeated measures ANOVA using SPSS 19.0 and for paired samples t-tests using G^*^Power 3.1.

### Result

**Figure 3:**
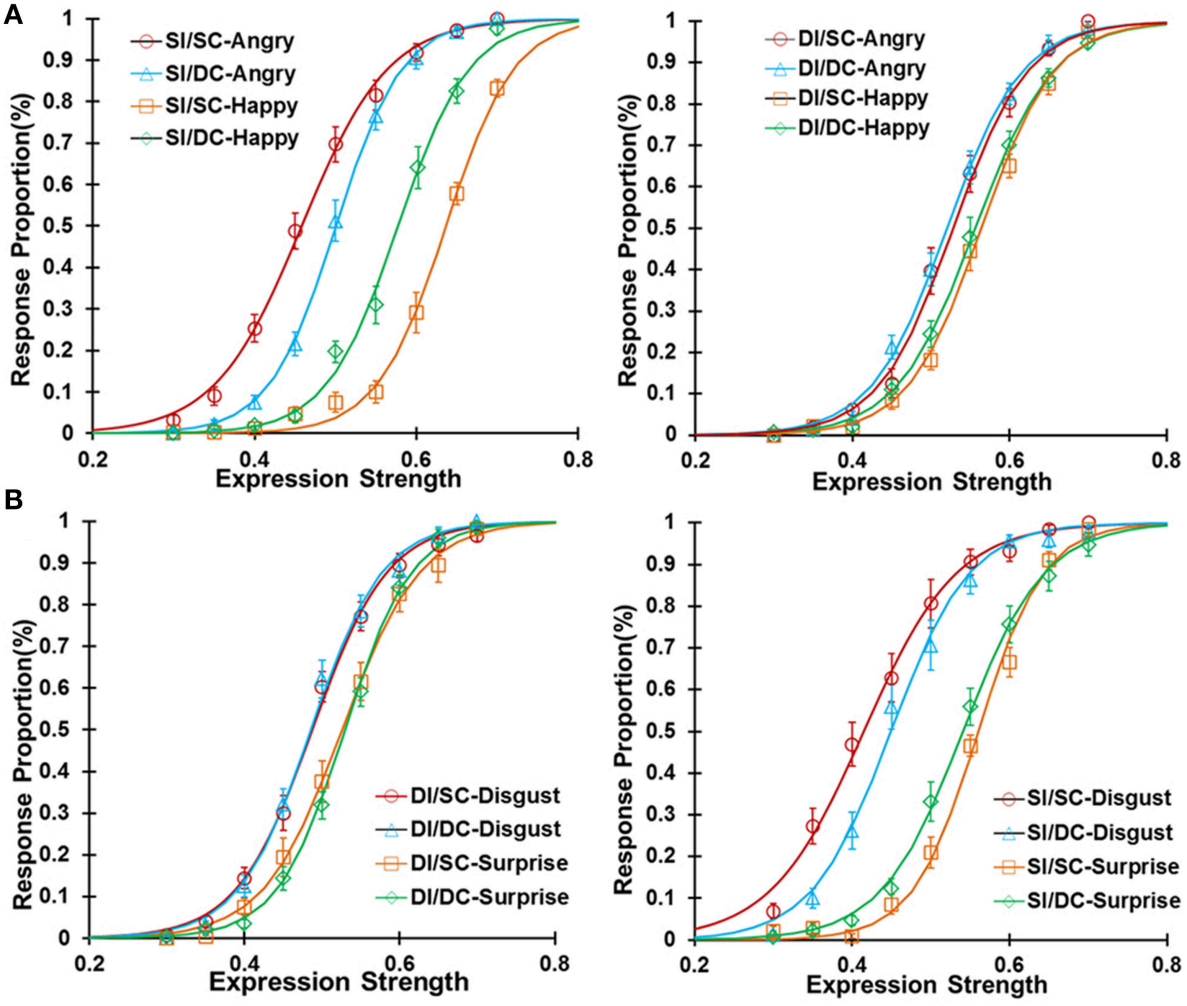
**The response proportion as a function of expression strength in Experiment 1 for the happy-angry expression pair (A) and the surprised-disgusted expression pair (B)**. The data were fitted with logistic functions averaged in the sixteen subjects.

**Figure 4:**
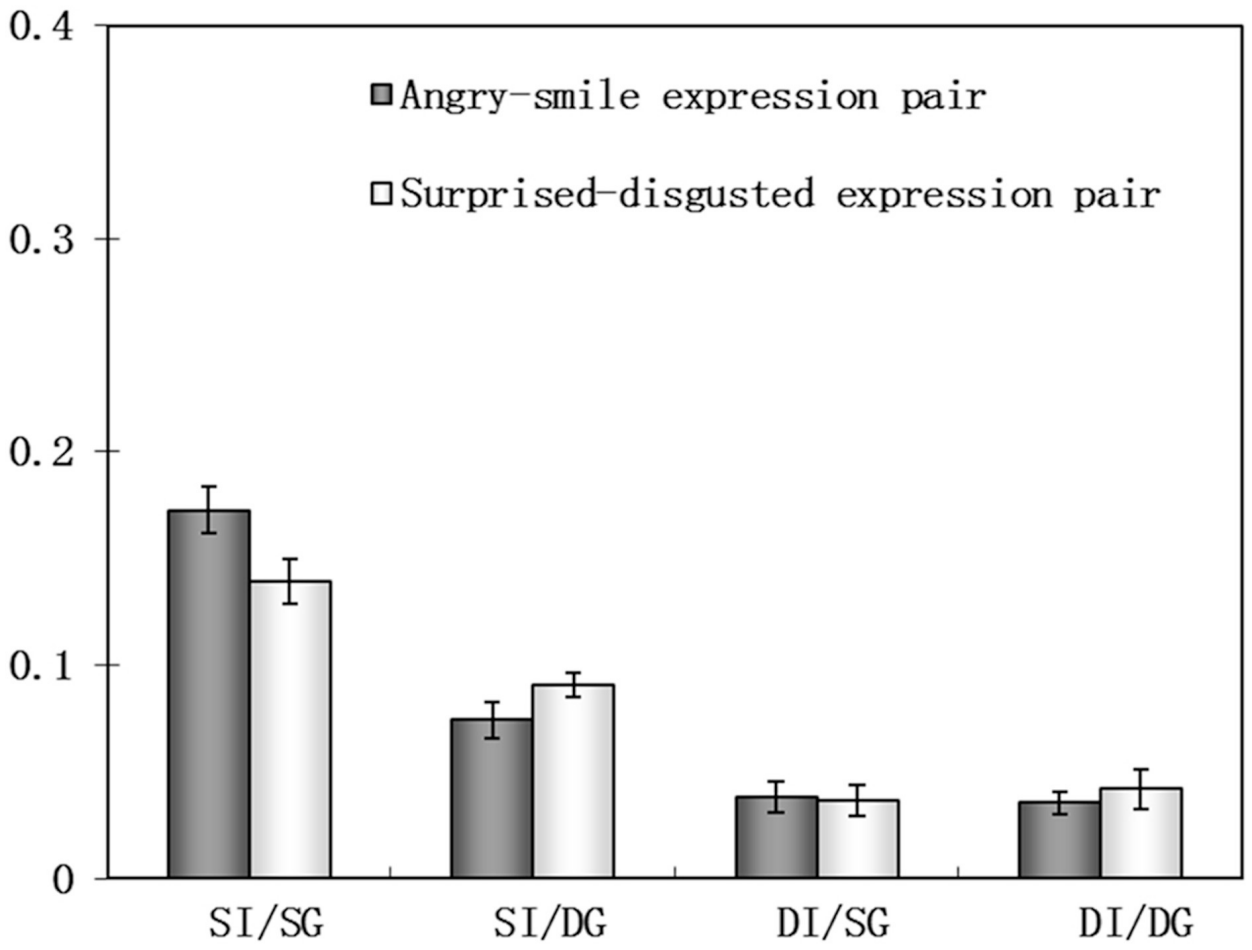
**The aftereffect magnitude in four adapting conditions in Experiment 1, bars indicate the magnitude of the expression aftereffect and the error bars denote SEM**.

We plotted the response proportions as a function of the expression strength of the test faces under four adapting conditions for happy-angry (Figure 3A) and surprise-disgusted expression (Figure 3B) pairs. All adapting conditions generated significant aftereffects (Figure 4, also see Table 1 for details). After adaptation to one expression, the subjects tended to see the test face as the other expression within an expression pair, and psychometric curve shifts to the opposite direction of the adaptor. These results confirmed the expression aftereffect reported in the previous literatures (Webster et al., 2004; Butler et al., 2008; Xu et al., 2012).

**Table 1:** **The expression aftereffect (Mean and SEM) for happy-angry and disgusted-surprised expression pairs in four adapting conditions**.

**Adapting conditions**	**Expression pairs**	**Mean (%)**	**SEM (%)**	**Statistic tests (*df* = 15)**
SI/SC	Happy-angry	17.26	1.08	*t* = 16.03, *p* < 0.001
	Disgusted-surprised	13.94	1.05	*t* = 13.32, *p* < 0.001
SI/DC	Happy-angry	7.41	0.81	*t* = 9.10, *p* < 0.001
	Disgusted-surprised	9.08	0.59	*t* = 15.35, *p* < 0.001
DI/SC	Happy-angry	3.81	0.69	*t* = 5.49, *p* < 0.003
	Disgusted-surprised	3.65	0.73	*t* = 5.04, *p* < 0.009
DI/DC	Happy-angry	3.54	0.54	*t* = 6.57, *p* < 0.002
	Disgusted-surprised	4.18	0.97	*t* = 4.33, *p* < 0.026

The result shows no significant main effect of the expression pair [*F*_(1, 15)_ = 0.21, *p* = 0.65], indicating that a similar expression aftereffect is obtained for both expression pairs. There were also no significant two or three interactions involving the variable of expression type. Consequently, the data were collapsed across expression pairs in further analyses.

The main effect of facial identity on expression aftereffect was significant [*F*_(1, 15)_ = 228.63, *p* < 0.001, power = 1.00], indicating smaller expression aftereffects when the adaptor and tests are different persons (*M* = 3.80%, SEM = 0.37%) compared to when they are the same person (*M* = 11.92%, SEM = 0.66%), averaged across two different expression configuration groups. This result is consistent with the observation that change in facial identity reduced the expression aftereffect, suggesting the dependence of expression representation on facial identity (Fox and Barton, 2007; Ellamil et al., 2008; Campbell and Burke, 2009; Vida and Mondloch, 2009; Pell and Richards, 2013).

There was a main effect of expression configuration on expression aftereffect [*F*_(1, 15)_ = 53.07, *p* < 0.001, power = 1.00]. We also found significant interaction between facial identity and expression configuration [*F*_(1, 15)_ = 29.05, *p* < 0.001, power = 0.99], suggesting that expression configuration influences the identity-dependent and identity-independent expression aftereffects in different ways. Simple effect analyses were performed to further explore the source of this interaction. The expression aftereffect in the same identity/different configuration condition (*M* = 8.25%, SEM = 0.52%) was much weaker than that in the same identity/same configuration condition (*M* = 15.60%, SEM = 0.80%) [*F*_(1, 15)_ = 71.13, *p* < 0.001, power = 1.00], suggesting change of expression configuration would impair the identity-dependent expression aftereffect. In contrast, the aftereffect magnitude in the different identity/same configuration condition (*M* = 3.73%, SEM = 0.49%) was approximately the same with that in the different identity/different configuration condition (*M* = 3.86%, SEM = 0.55%) [*F*_(1, 15)_ = 0.02, *p* = 0.88, power = 0.046]. This indicates that the identity-independent expression aftereffect is robust to the variance in expression configuration in comparison to identity-dependent expression aftereffect. It should be noted that the statistical power for this contrast is relatively low, and one may argue that the robustness of identity-independent is simply due to statistical error. A control experiment is required to exclude this possibility (see Experiment 2). Finally, the expression aftereffect in different identity/same configuration condition was weaker than that in same identity/same configuration condition [*F*_(1, 15)_ = 59.98, *p* < 0.001, power = 1.00], indicating that the reduction of expression aftereffect across identities is held even when the adaptor and test face has the same expression configuration. This suggested the reduction of cross-identity expression aftereffect cannot completely be attributed to change in expression configuration, confirming further the role of facial identity in expression aftereffect.

## Experiment 2

Although Experiment 1 shows no significant difference in aftereffect size between different identity/different configuration and different identity/same configuration conditions, indicating identity-dependent expression aftereffect is robust to expression configuration change. The statistical power for this observation is, however, not high, which is due to the interference of identity-dependent expression representation. Here, we further confirmed this observation using schematic line face as the adaptor and real faces as tests. It is well established that the adaptation effect is modulated by the perceptual similarity between adaptor and test. As the line face is totally dissimilar with real face and thus only conveys the emotional information of an expression, using the line face as adaptor could exclude the possible contribution from the identity-dependent neural representation to expression aftereffect. Besides, the line face enables us to manipulate more precisely the expression configuration and/or expression intensity. If the identity-independent expression aftereffect is robust to the variance in expression configuration, it can be predicted that the magnitude of the expression aftereffect should be approximately equal, even with different expression configurations. Otherwise, the aftereffect magnitude should differ significantly from different expression configurations.

### Subject

The subjects were twenty paid students (Mean age: 22.5, *SD* = 2.9) from Shanghai Maritime University, and each completed one 40 min sessions. All subjects have normal or corrected-to-normal vision, and all were naïve to the aims and purpose of the experiment.

### Stimuli and apparatus

As the line faces cannot vividly express complex negative expressions, such as surprise or disgust, we used happy and sad expressions in Experiment 2. The four line faces displaying happy or sad expressions were created using Adobe Photoshop CS 6.0. These line faces were made of an ellipse with white background for the face area, two black dots for eyes, and a curvature for the mouth. The eyes and mouth are located at one-third and two-thirds of the major axis of the face, respectively. The center-to-center distance between the two eyes is one-third of the minor axis of the face. We gradually changed the curvature of the mouth from concave to convex to express a happy or sad expression.

**Figure 5:**
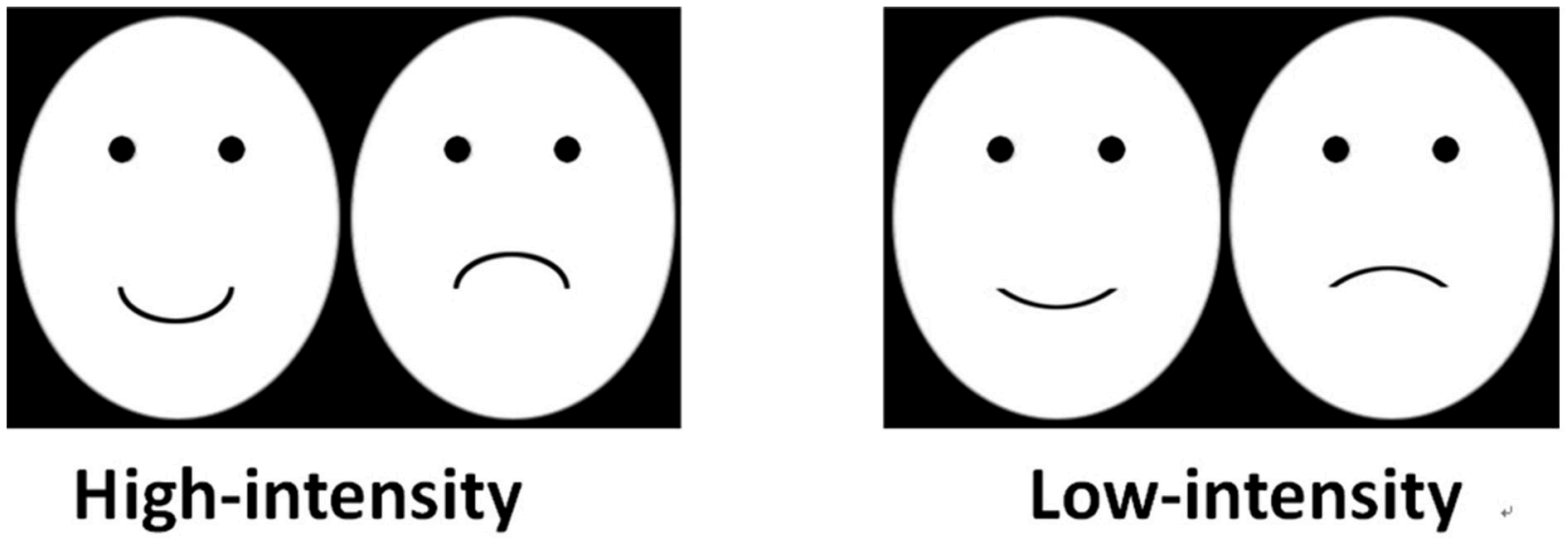
**The line faces as adaptors used in Experiment 2**.

There were two adapting conditions in Experiment 2, in which faces having mouths with large curvatures were used as adaptors for high-intensity adapting condition, while those with small curvatures were used as the adaptors for low-intensity adapting condition (Figure 5). Ten subjects were instructed to rate the expression intensity on these adapting faces using Likert method (Likert scale is from 1 to 9). As expected, the happy face having large curvature (*M* = 6.9, SEM = 0.48) presented higher expression intensity than that having small curvature (*M* = 3.8, SEM = 0.49) [*t*_(9)_ = 6.15, *p* < 0.001, power = 1.00], and the sad face having large curvature (*M* = 6.1, SEM = 0.50) is also higher in expression intensity than that having small curvatures (*M* = 4.1, SEM = 0.35) [*t*_(9)_ = 7.75, *p* < 0.001, power = 1.00].

For these two conditions, the adaptors were different but the tests were the same. The male photographic subject, displaying the happy and sad expressions, was selected from the affiliated image set of Facial Action Coding System to construct the test faces. Similar to Experiment 1, we created the ambiguous expression images with sadness strength from 0% (happiest) to100% (saddest) in steps of 5% using Abrosoft FantaMorph 5.0, and nine faces with sadness strength from 30 to70% were used as test faces. The image size, displaying apparatus, and presenting method were the same as in Experiment 1.

### Procedure

The procedure is identical to that of Experiment 1 with the following exceptions. First, the adaptors were computer-generated line faces instead of real faces. Second, we randomly interleaved the catch trials using the inversion of cartoon faces or real faces as the adaptation stimuli, which was to prevent subjects from simply using the local mouth area instead of whole face to recognize the expression (Xu et al., 2008). These catch trials were not further analyzed.

### Result

**Figure 6:**
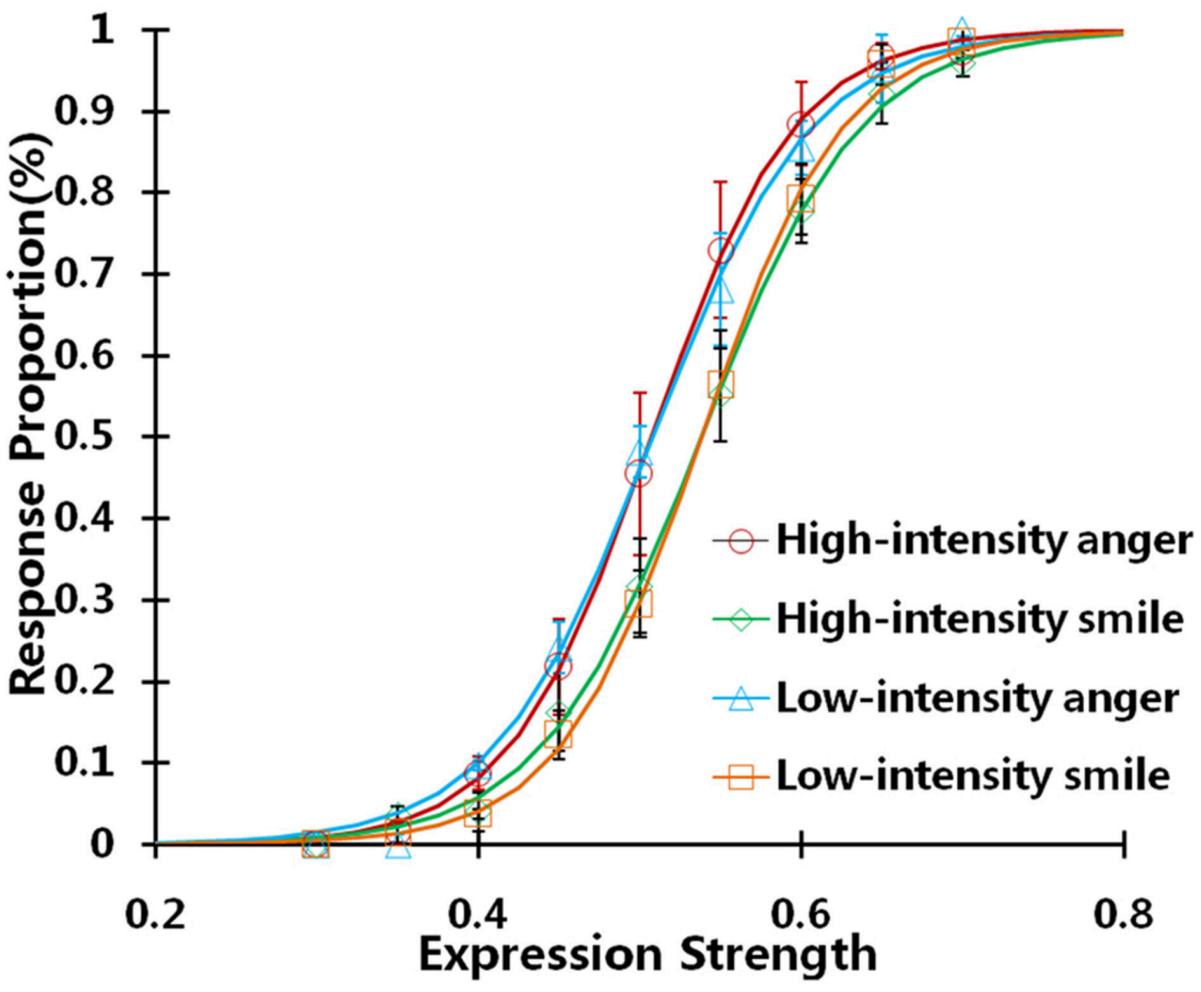
**The response proportion as a function of the expression strength in the Experiment 2**. The data is fitted with logistic functions averaged in all twenty subjects.

We plotted the response proportions as a function of the expression strength of the test faces under two adapting conditions (Figure 6). The line faces generated significant aftereffects on two adapting conditions, although the aftereffect strength is relatively weak compared to that induced by the real face as the adaptor in Experiment 1. The results are consistent with prior studies (Butler et al., 2008; Xu et al., 2008), which found that a simple curvature and a cartoon face can generate the observable aftereffect on real face.

The aftereffect magnitude was 2.89% (SEM = 0.57%) in the high-intensity adapting condition, and 2.60% (SEM = 0.78%) in the low-intensity adapting condition. A paired sample t-test showed no significant difference between these two conditions [*t*_(19)_ = 0.383, *p* = 0.706, power = 0.92]. The observation is similar to the result obtained from the real face in Experiment 1, which provided strong evidence suggesting the identity-independent expression aftereffect is robust to the change in the expression configuration in comparison to identity dependent expression aftereffect.

## Discussion

Our results first confirmed that the expression aftereffect is reduced when the facial identity is manipulated, a finding that several studies have observed before. This observation does not relate to the specific expression pairs, as the similar expression aftereffect pattern was observed in both the happy-angry and in surprised-disgusted expression pairs. Campbell and Burke (2009) also found that the extent of the aftereffect reduction across identities was almost the same for five basic emotional expressions, although the processing of these different expressions appears to involve different neural mechanisms and visual areas (e.g., Posamentier and Abdi, 2003). This suggests the processing of individual facial expressions depend on facial identity similarly.

The reduction in the cross-identity expression aftereffect is not simply due to change in expression configuration between adaptor and test faces, although a change in expression configuration does influence expression aftereffect. The result in Experiment 1 shows that the expression aftereffect is still reduced even when the adaptor and test faces have identical expression configurations, suggesting the functional influence from facial identity on expression aftereffect. Such observation is consistent with Ellamil et al.'s (2008) work, which used artificial faces of angry and surprised expressions as adaptors and found similar reduction in the adaptation effect when the adaptor and test faces had the same expression morphing prototype but different facial textures and contours. Together, our and Ellamil et al.'s (2008) observation consolidates Fox's proposal of the identity dependent neural representation.

We further determined how a change in the expression configuration influences the identity- dependent and identity-independent expression aftereffects, respectively. This was achieved using real faces as adaptors in Experiment 1 and using line faces as adaptors in Experiment 2. The results were consistent in two experiments. Experiment 1 found that a change in expression configuration impaired the identity-dependent expression aftereffect, but not the identity-independent expression aftereffect. The Experiment 2 further confirmed the observation of Experiment 1 using the line faces as adaptor. We found that the identity-independent expression aftereffect is consistently robust to the variance in the expression configuration relative to the identity-independent expression aftereffect.

Why do identity-dependent and identity-independent expression systems show different sensitivities to expression configuration? The possible explanation is that these two systems depend on the different facial components to process emotional expressions. As the structural reference hypothesis stated (Ganel and Goshen-Gottstein, 2004), the face structure information is not only important to identity discrimination, but it is also used by observers as a reference to compute and recognize expressions. The identity-dependent expression system may rely more on the facial shape and/or structure information (e.g., local edge, facial contour) to perform an expression configuration analysis, thus it is sensitive to change in spatial configurations of expressions. In support of this notion, Neth and Martinez (2009) have demonstrated that simple structural changes in emotionless faces could induce the virtual perception of facial expression, with shorter vertical distance between eyes and nose resulting in the perception of anger, and the larger distance leading to the perception of sadness. On the other hand, the identity-independent expression system seems to depend on the information of emotion category; thus, it is more robust to the detailed variance in the configuration properties of the face. Just as in daily life, although the happy expression can be displayed with different configurations or different intensities in the face, all these expressions would be perceived as a happy emotion within a certain range of expression intensities and configurations. It is possible for the identity-dependent expression system to interpret the spatial configuration of an expression, as well as for identity-independent neural system to receive the input of identity-dependent expression system and extract the emotional information from the facial expression.

There is a long-term debate whether facial expressions are represented as belonging to discrete categories of emotion or as continuous dimensions (see Bruce and Young, 2012; Harris et al., 2012). The category model is supported by the evidence that faces within a category were discriminated more poorly than faces in different categories that differed by an equal physical amount (Etcoff and Magee, 1992; Calder et al., 1996). In contrast, the observation that human can accurately perceive differences in intensity (Calder et al., 1996) and variation (Rozin et al., 1994) of a given emotional expression is consistent with the continuous model. Our result supports a synthesis of the above two models by showing that expression is represented in both a categorical and a continuous ways. Specifically, the identity-independent expression system is robust to variance in expression configurations within same emotion category, suggesting it may perceive emotional expressions in a category manner; In contrast, the high sensitivity to expression configuration supports a continuous manner in the identity-dependent expression system.

We suggest two possible candidates of brain areas responsible for identity dependent expression representation. One possible candidate of brain area responsible for the identity-dependent expression representation is the posterior superior temporal sulcus (STS). A recent fMRI adaptation study investigated adaptation to both facial identity and expression (Winston et al., 2004). Adaptation to identity but not expression was found in the fusiform face area, and adaptation to expression but not identity was found in the middle STS. In the posterior STS, there were large adaptation effects to identity and a smaller adaptation effect to expression. These observations suggest that the posterior STS area may encode both identity and expression. Future researches are desirable to examine whether the adaptation effect is sensitive to the change in expression configuration in the same person in the posterior STS. The other candidate for the identity-dependent expression representation is the FFA. Although this area is generally believed to be responsible for the processing of static information of the face in identity discrimination (Haxby et al., 2000; Winston et al., 2004), the FFA is also found to be sensitive to variations in expression even when attention was directed to identity (Ganel et al., 2005). The findings fit with our behavior data and suggest an interactive system for the processing of expression and identity in the FFA area.

It is relatively difficult to infer the possible brain area related to identity-independent expression representation. Although Winston et al. (2004) found that repeating emotional expressions across different face pairs led to reduced signal in the middle STS, showing the identity-independent processing of expressions in the middle STS. However, the middle STS is sensitive to the dynamic and transient changes in facial features, which contrasts with the robustness of the identity-independent expression aftereffect to expression configuration observed in current study. We suggest that the amygdala is a possible candidate. This area is highly related to the processing of emotional expressions, and has previously found to be insensitive to expression change within the same emotion category (Harris et al., 2012), which well fits with our result observed in identity-independent expression aftereffect. On the other hand, it is worth pointing out that the amygdala may be not the only area involved in identity-independent processing of expressions. As amygdala is generally related to processing of fear and happy expressions (Morris et al., 1996; Blair et al., 1999), and the other expressions, such as disgusted expression, could not induce the activation in the amygdala (Phillips et al., 1997). The disgusted expression, however, shows the similar result as the fear expression in first experiment. This appears to suggest that other brain areas besides amygdala were also involved in identity-independent expression processing. It would be interesting for future studies to investigate whether the activation of the other expression-related brain areas are robust to the change in expression configuration using an fMRI adaptation paradigm.

### Conflict of interest statement

The authors declare that the research was conducted in the absence of any commercial or financial relationships that could be construed as a potential conflict of interest.

## Footnotes

^1^The JNDs of Caucasian and Asian faces were measured using the Method of Adjustment (Gescheider, 1997). Two Asian faces displaying a happy or an angry expression were selected from the CAS-PEAL Face Database (Gao et al., 2008), and visually matched in expression intensity with Caucasian faces. We generated 101 images with the proportion of happiness from 0% (happiest) to 100% (angriest) in steps of 1% for Asian and Caucasian faces, respectively. Within these expression images, the expression images with the proportions equal to 20, 40, 50, 60, 80% were used as the references. In each trial, the test face and one of references were presented side by side at the center of the screen, and the subject was instructed to adjust the expression strength of test face by step of 1% so that it is perceptually identical to the reference face. The standard deviation of the distribution of the adjustments within 20 trials is taken as an estimate of the difference threshold (JND) for each reference level. The JND averaged across five reference levels were 6.72% (SEM = 0.33%) for Caucasian face and 6.56% (SEM = 0.40%) for Asian faces. The Two-way repeated measures ANOVA with reference level and race as within-subject factors shows no main effect of race [*F*_(1, 5)_ = 0.78, *p* = 0.42] and interaction between race and reference level [*F*_(4, 20)_ = 2.12, *p* = 0.11].
